# A Modified GC-MS Analytical Procedure for Separation and Detection of Multiple Classes of Carbohydrates

**DOI:** 10.3390/molecules23061284

**Published:** 2018-05-26

**Authors:** Yong-Gang Xia, Hui-Min Sun, Tian-Long Wang, Jun Liang, Bing-You Yang, Hai-Xue Kuang

**Affiliations:** Key Laboratory of Chinese Materia Medica, Heilongjiang University of Chinese Medicine, Ministry of Education, Harbin 150040, Heilongjiang, China; yonggangxia@163.com (Y.-G.X.); hhuiminsun@163.com (H.-M.S.); wtl890322@163.com (T.-L. W.); lliangjunn@163.com (J.L.); ybywater@163.com (B.-Y.Y.).

**Keywords:** GC-MS, trimethylsilyl-dithioacetal, ketose, amino sugar, aldoses, uronic acids

## Abstract

A modified GC-MS analytical procedure based on trimethylsilyl-dithioacetal (TMSD) derivatization has been established for a simultaneous determination of thirteen carbohydrates. Different from previous approaches, the current GC-MS method was featured by a powerful practicability for simultaneous detection of aldoses, uronic acids, ketoses, and amino sugars; simplifying GC-MS chromatograms and producing a single peak for each derivatized sugar, as well as high resolution, sensitivity, and repeatability. An additional liquid-liquid extraction from derivatization mixtures was performed not only to increase the detection sensitivity of amino sugars but also to decrease the by-products of derivatization. Contrarily, three amino sugars were detected at a very low intensity or not detected at all. The effect of time on monosaccharide- mercaptalated reaction was systematically investigated. The effect of trimethylsilylation on the formation of TMSD was also optimized. The established GC-MS based on TMSD derivatization was suitable for complex carbohydrate analysis and has been successfully applied for the detection of free carbohydrates in water extracts of *Anemarrhena asphodeloides* roots and determination of monosaccharides in *Glossy ganoderma* polysaccharides.

## 1. Introduction 

Carbohydrates from plants, especially oligosaccharides and polysaccharides, have attracted more attention in recent decades because of their biological functions, as well as their roles as structural materials and energy sources [1,2]. In addition, the presence of stereoisomers or similar structures requires high-resolution separation and determination techniques for carbohydrate analysis. High-performance GC-MS procedure is the technique for carbohydrate analysis, because of the high-resolution separation and sensitivity of detection [3,4]. It is well-known that many GC-MS methods have been applied to analyze carbohydrates such as methyl ethers [5], acetates [6], trifluoroacetates [7], trimethylsilyl ethers (TMS) [8], TMS oximes [9], and diethyl dithioacetal [10]. However, some methods were only limited to several monosaccharides or low derivatization yields, complicated chromatograms, and time-consuming and inseparable chromatographic peaks [4,10,11].

In this study, a precolumn derivatization based on trimethylsilyl-dithioacetal (TMSD) derivatives has been established, employing GC-MS for a simultaneous analysis of multiple classes of carbohydrates. These carbohydrates were characterized by the simultaneous presence of seven aldoses, one ketose, two uronic acids and three amino sugars. Furthermore, the chromatographic system can provide retention data which serve as complementary information for the positive identification of resolved carbohydrates. The established GC-MS method, based on TMSD derivatization, was suitable for the complex carbohydrate analysis and has been successfully applied for free carbohydrates in water extracts of *Anemarrhena asphodeloides* and monosaccharide compositions in *Glossy ganoderma* polysaccharides.

## 2. Experimental Section

### 2.1. Materials and Reagents

Both *A. asphodeloides* and *G. ganoderma* were provided by First Affiliated Hospital of Heilongjiang University of Chinese Medicine. d-galacturonic acid (GalUA), d-glucuronic acid (GlcUA), d-fucose (Fuc), l-rhamnose (Rha), d-glucose (Glc), d-galactose (Gal), d-mannose (Man), l-arabinose (Ara), d-fructose (Fru), d-xylose (Xyl), d-glucosamine hydrochloride (GlcN), d-galactosamine hydrochloride (GalN), d-mannosamine hydrochloride (ManN), meso-erythritol (Ery), hexamethyl disilazane (HMDS) and trimethylchlorsilane (TMCS) were purchased from Sigma-Aldrich (St. Louis, MO, USA). Trifluoroacetic acid (TFA) was purchased from Merck (Zdarmsta, DT, Germany). Water was obtained from a Milli-Q purification system (Millipore, Bedford, MA, USA). All other chemicals were of the highest analytical grade.

### 2.2. Preparation of Water Extracts of A. asphodeloides

The dry sample materials (5 g) were extracted three times, with 10-fold the volume of distilled water under reflux for two hours each time. After filtering, the obtained extract was condensed under vacuum until getting a syrup. The crude syrup was further lyophilized to yield the water extract of *A. asphodeloides*.

### 2.3. Preparation of G. ganoderma Polysaccharides and Complete Acid Hydrolysis 

The dried sample materials (5 g) were extracted 3 times, with 10 folds of the volume of distilled water under reflux for 2 h each time. The filtrate of the obtained extract was condensed under vacuum and precipitated with 75% ethanol. After standing for 24 h at 4 °C, the precipitated was collected and washed with anhydrous ethanol, then dried. The residue was redissolved in water, and after centrifugation (4000 rpm for 15 min) the supernatant was dialyzed (cut-off M_w_ 3500 Da) against tap water and distilled water for 48 h, and lyophilized to yield crude *G. ganoderma* polysaccharides. 

The crude polysaccharides (6 mg) were further treated with 3 mL of 2 M TFA in a pocket flask. The suspension was incubated at 110 °C for 2 h. After hydrolysis, the hydrolysate was washed with methanol and evaporated to dryness several times to remove the residue of TFA. The sample, hydrolyzed and dried, was added 1.0 mL distilled water and centrifugated at 12,000 rpm for 10 min. The supernatant was transferred to a centrifuge tube of 2 mL and diluted with deionized water and stored in 4 °C.

### 2.4. TMSD Derivatization of Carbohydrates

TMSD derivatization was performed on previous reports with appropriate modifications [4,10]. Briefly, a mixture of ethanethiol and TFA (2:1) was added to standards or samples, and the reaction vessel was closed tightly with a screw cap. The residue in the vial was dissolved by swirling and the resulting solution was kept for 10 min at 25 °C. Natural dried residues were added into 100 μL of pyridine, 68 μL of HMDS, and 22 μL of TMCS. The resulting mixed solution was further heated for 30 min at 70 °C in a water bath. Additionally, appropriate amounts of both water and chloroform were added into this mixed solution for liquid–liquid extractions. The chloroform solution was directly filtered through a 0.22 μm membrane before GC-MS analysis.

### 2.5. GC-MS Apparatus and Conditions

GC-MS analyses were performed on an Agilent 7890A GC system equipped with a 5975C EI-MS system (Agilent Technologies, CA, USA). The chromatographic separation was conducted on a DB-5 silica capillary column (60 m × 0.25 mm × 0.25 μm) using the following temperature profile: 80 °C for 0 min, 80–190 °C at 2.5 °C/min, 190–252 °C at 2 °C/min, 252–300 °C at 25 °C/min, 300-310 °C at 25 °C/min and held for 15 min. The ionization was performed in the electron impact mode at 70 eV. The ion source temperature was 230 °C, and the interface temperature was 250 °C. Mass spectra were recorded both in total ion chromatogram (TIC) mode (*m/z* 50-550) and selected ion monitoring (SIM) modes. These SIM ions were: *m/z* 249 and 319 for Xyl and Ara; *m/z* 249 and 333 for Rha and Fuc; *m/z* 135 and 305 for GalUA and GlcUA; *m/z* 307, 319 and 331 for Glc, Man and Gal; *m/z* 217 and 361 for Fru; *m/z* 131 and 204 for GlcN, GalN and ManN; and *m/z* 217 and 307 for Ery.

### 2.6. Method Validation

The method was validated for linearity, limit of detection (LOD), limit of quantitation (LOQ), precision, and accuracy. All calibration curves were constructed from peak areas of reference standards versus their concentrations. A water stock solution of thirteen reference standards was prepared as the final concentrations (μg/mL) ascribed to 3254.1 for GlcN, 3296.2 for GalN, 3263.6 for ManN, 6641.5 for Fru, 1682.4 for Xyl, 1532.9 for Ara, 3360.7 for Rha, 3381.1 for Fuc, 4152.4 for GalUA, 4102.8 for GlcUA, 2014.3 for Glc, 2037.6 for Man, and 2016.4 for Gal; thus, it was diluted to appropriate concentrations to establish calibration curves. The LOD and LOQ under the chromatographic conditions were determined at an S/N of 3 and 10, respectively. The recovery was tested by spiking a solution containing known quantities of the standard into known amounts of powdered *A. asphodeloides* samples, mixed prior to extraction. The recovery was calculated as follows: recovery (%) = 100 × (amount found – original amount)/amount spiked.

## 3. Results and Discussion

### 3.1. Optimization of Derivatizations by Formation of TMSDs

Although some studies have described the optimized derivatization reactions on the formation of TMSDs, these derivatization parameters were only focused on aldoses and uronic acids [4,10]. Aldoses and uronic acids were transformed into the corresponding diethyl dithioacetal aldehydes and diethyl dithioacetal lactones, respectively. However, this study showed to be more complex than previous ones due to additional occurrences of one ketose and three amino sugars. Therefore, simultaneous optimization regarding the production of optimal TMSD derivatives is necessary for an accurate analysis of these thirteen compounds. The effects of time on the monosaccharide- mercaptalated reaction were systematically investigated as the first step. The TMS reaction of monosaccharide-mercaptalated derivatives on the formation of TMSD was further optimized.

**Figure 1:**
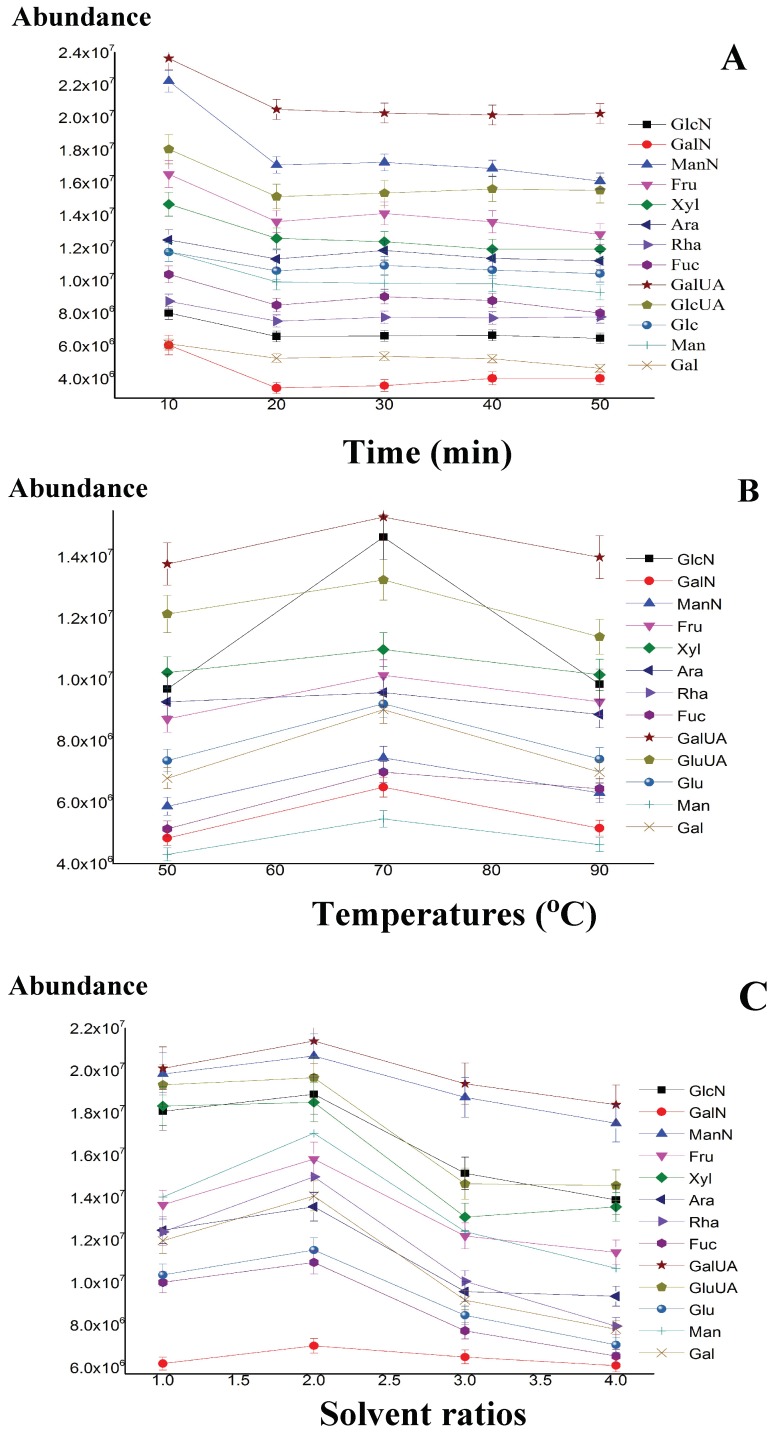
Effects of derivatization time on monosaccharide-mercaptalated (**A**), effects of both TMS derivatization temperatures (**B**), and solvent ratios between HMDS and TMCS (**C**) on formations of TMSDs.

The effects of time on the monosaccharide-mercaptalated reaction were examined using the following gradient program at 10, 20, 30, 40 and 50 min with HMDS and TMCS 68:22. Figure 1A shows that the thiolation derivatization reaction time for all thirteen carbohydrates was determined to be 10 min, as the time increase, the peak areas of the investigated thirteen compounds had not been changed significantly. This fact indicated that both the ketose and amino sugars produced the same mercaptalated derivatization yields, as those of both aldoses and uronic acids [10]. 

The TMS reaction temperatures on the formation of TMSD derivatives are described in Figure 1B. The temperatures of the peak response values were examined by using the following gradient program at 50, 70 and 90 °C, the other two variables were set as follows: reaction time 10 minutes, and solvent ratios between HMDS and TMCS as 68:22. The results showed that the temperature was set at 70 °C, which facilitated the maximum TMS reactions for all thirteen carbohydrates.

Different ratios between HMDS and TMCS (solvent amount using HMDS and TMCS) are also an important factor to affect the TMS reaction completely. Briefly, solvent ratios between HMDS and TMCS were set at 68:22, 132:44, and 264:88 when the reaction time and reaction temperatures were held for 10 minutes at 70 °C. As shown in Figure 1C, the highest derivatization of efficiency was obtained for all seven aldoses, one ketose, two uronic acids and three amino sugars with a solvent ratio of 68:22 between HMDS and TMCS. Since it is difficult to prepare precise amount sample preparations because of the required derivatization procedures, the internal standard method was used and Ery was chosen to be the internal standard.

### 3.2. Modification of Sample Preparation of TMSD Derivatives 

**Figure 2:**
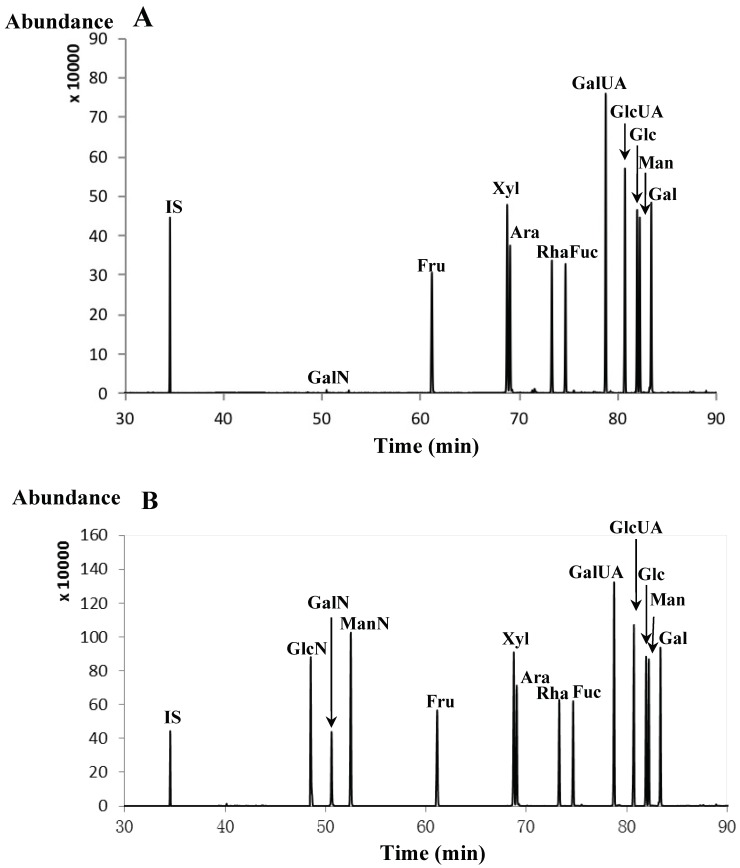
GC-MS chromatograms of TMSD derivatives of monosaccharide standards without and with chloroform liquid-liquid extractions to give (**A**) and (**B**), respectively. The sample concentration was 3/5 of the mixed reference stock solution, as shown in Section 2.6.

In order to increase sensitivity and specificity in detection, the selection of ion detection (SIM) of each TMSD was performed by GC-MS. After appropriate modification of chromatography conditions based on known methods [12], the optimal GC-MS conditions were shown in Section 2.5. However, as shown in Figure 2A, the three amino sugars showed both low sensitivity and poor repeatability using previous sample preparation methods [10]. In contrast, an additional liquid-liquid extraction from derivatization mixtures using chloroform was performed to solve this problem (Figure 2B). The results confirmed that this additional liquid-liquid extraction from derivatization mixtures played an important role not only to increase the detection sensitivity of amino sugars but also to decrease of by-products of derivatization. Thus, the complete baseline separation of all corresponding isomeric carbohydrate derivatives are shown in Figure 2B; including *m/z* 131 and 204 for **1**-**3** (GlcN, GalN, and ManN); *m/z* 217 and 361 for **4** (Fru); *m/z* 249 and 319 for **5** and **6** (Xyl and Ara); *m/z* 249 and 333 for **7** and **8** (Rha and Fuc); *m/z* 135 and 305 for **9** and **10** (GalUA and GlcUA); and *m/z* 307, 319 and 331 for **11**-**13** (Glc, Man, and Gal). Besides, *m/z* 217 and 307 were used for the internal standard of Ery. It is worth mentioning that this is the first example on simultaneous analysis of three amino sugars (**1**–**3**), one ketose (**4**), seven neutral carbohydrates (**5**-**8** and **11**-**13**) and two uronic acids (**9** and **10**) using GC-MS technique based on TMSD derivatizations. Table 1 summarized fragmentation patterns and retention index (RI) values of all compounds tested. For the determination of RI values of TMSDs, C_7_-C_40_ alkane mixture in hexane was separated by the same GC-MS chromatographic condition. The RI values of aldoses and uronic acids are well matched with those of reference standards reported in the literature [10].

### 3.3. Method Validations

The analytical procedure has been validated to confirm its reliability. Table 2 summarizes the calibration curves, LOD and LOQ values, of the carbohydrate analyzed by GC-MS. All the peaks showed good linearity (*R*^2^ > 0.99) in a wide concentration range. The LOD and LOQ were 3 and 10, respectively; they were determined from signal-to-noise (S/N) ratio. The results showed that the LODs and the LOQs of monosaccharides were in the range of 0.6–2.7 μg/mL and 3.1–13.3 μg/mL, respectively, indicating that the sensitivity of the method was satisfactory. For stability test, the same sample solution was analyzed for thirteen analytes every 4 h in 24 h with RSD less than 2.02%; which indicated that the sample was stable over 24 h under experimental conditions. Furthermore, recovery experiments were performed to investigate the accuracy of this method. A known amount of the corresponding carbohydrates (approximately 100% of the content, *n* = 3) were added into the sample of *A. asphodeloides*, and the resulting sample was subjected to the entire analytical sequences. The recovery rate of this method was 95.17%–106.02% and RSD less than 2.02%, suggesting that this method is accurate and practical for the free carbohydrate analysis in *A. asphodeloides*.

### 3.4. Application to Real Samples

**Figure 3:**
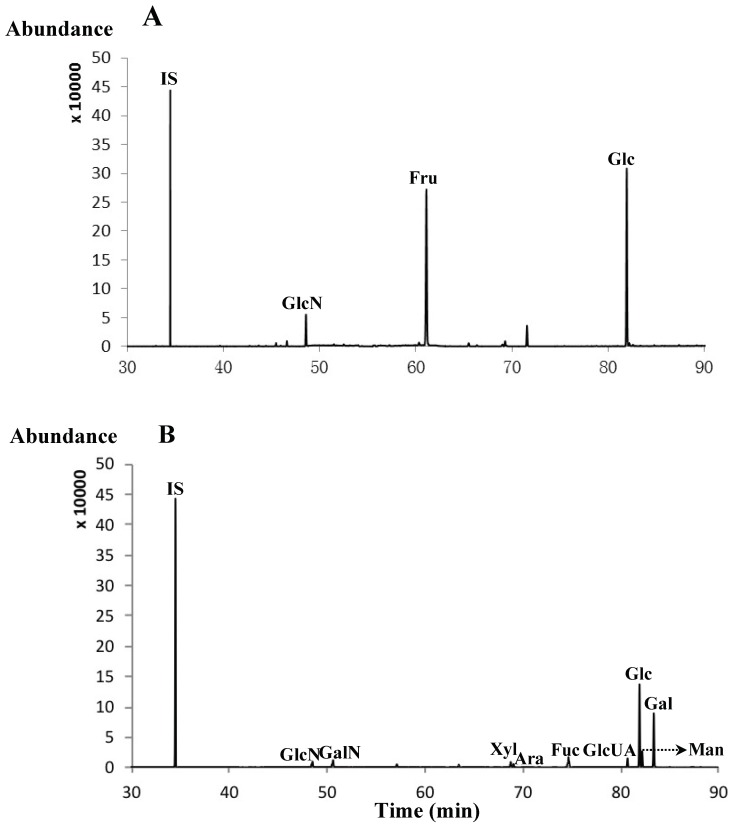
GC-MS chromatograms based on TMSD derivatives for free carbohydrates in the water extracts of *A. asphodeloides* (**A**) and full acidic hydrolysis products of *G. ganoderma* polysaccharides (**B**).

Our previous reports showed that the water extracts of *A. asphodeloides* possessed anti-inflammatory, immunomodulatory and to improve the laxative effects in the bowel [13]. The steroidal saponins, lignans and polysaccharides have been confirmed to be major structural types of biological constituents in the water extracts of *A. asphodeloides* [14,15]. However, many free carbohydrates were also found by thin-layer chromatography methods. In order to investigate the species and contents of free carbohydrates in the water extracts of *A. asphodeloides*, the established GC-MS method, based on TMSD derivatizations, was further performed in this study (Figure 3A). It was found that the predominant free carbohydrates were Glc and Fru along with a small amount of GlcN in water extracts of *A. asphodeloides*; their corresponding molar ratios were 50.23:46.12:3.65.

On the other hand, it has been reported that *G. ganoderma* polysaccharides have strong antioxidant [16], immunomodulating [17], antitumor activities [18], etc. A study has been performed using HPLC-PMP derivatizations to determine complex monosaccharide compositions in *G. ganoderma* polysaccharides [19]. Here, the developed GC-MS method, using TMSD derivatizations, has been successfully applied to determine the major monosaccharides liberated from *G. ganoderma* polysaccharide samples. After TMSD derivatizations of *G. ganoderma*, polysaccharides were treated by 2M TFA to complete acid hydrolysis, the resulting sample was directly injected and separated under the optimum condition shown in Figure 3B. It was clear that the major monosaccharides in *G. ganoderma* polysaccharides were Fuc (**8**), Glc (**11**), Man (**12**), and Gal (**13**), along with some minor sugars such as GlcN (**1**), GalN (**2**), Xyl (**5**), Ara (**6**), and GlcUA (**10**). For all these polysaccharides, their corresponding molar ratios were 7.30:44.78:8.08:30.02:1.81:2.08:1.53:1.59:2.81, respectively. The results indicated that the monosaccharide compositions in *G. ganoderma* were in agreement with previous reports [19]. 

Based on above evidence, GC-MS coupled with modified sample preparation approach for TMSD derivatizations is a reliable and comprehensive method for the simultaneous detection and separation of multiple classes of carbohydrates, including aldoses, uronic acids, ketose and amino sugars. Thus, this GC-MS strategy is suited for complex carbohydrate analysis in various matrixes.

## 4. Conclusions

In this work, a reliable, simple and sensitive GC-MS method, based on modified sample preparation for TMSD derivatizations, was developed for the simultaneous analysis of thirteen carbohydrates. These thirteen monosaccharides were structurally characterized as seven aldoses, one ketose, two uronic acids and three amino sugars. Here we have to admit that this method has some limitations as the previous methods due to long run time, as well as the use of thiol as regent of derivatization [10]. However, except for its disadvantages, this modified GC-MS strategy should be focused on its prominent advantages. By comparison with other common methods, such as direct TMS derivatization and aldiol acetates [6,8], major merits of the current method could be summarized as followed: (1) powerful practicability for simultaneous detection of aldoses, uronic acids, ketose and amino sugars; (2) simplified GC-MS chromatograms obtained by producing a single peak for each derivatized sugar; and (3) great simplicity as well as good sensitivity and repeatability. 

## Figures and Tables

**Table 1:** Fragmentation patterns and RI values of all TMSDs.

No.	TMSDs	RI	*m/z* (Intensity, %)
1	GlcN	1836	73(47.7), 131(100), 147(15.9), 191(11.7), 204(22.1), 217(10.5), 259(3.5), 361(2.5)
2	GalN	1873	73(47.7), 131(100), 147(11.9), 191(7.0), 204(8.1), 217(8.1), 259(1.1), 361(3.0)
3	ManN	1906	73(45.8), 131(100), 147(15.6), 191(7.8), 204(16.0), 217(8.9), 259(1.8), 361(4.0)
4	Fru	2020	73(100), 103(9.5), 147(46.0), 191(20.5), 217(30.0), 243(23.6), 361(97.9)
5	Xyl	2125	73(100), 103(22.0), 135(25.7), 147(48.1), 205(61.8), 249(74.0), 307(5.8), 319(77.8)
6	Ara	2129	73(100), 103(20.1), 135(25.9), 147(45.8), 205(56.2), 249(80.0), 307(6.0), 319(69.9)
7	Rha	2196	73(100), 117(81.6), 135(30.2), 147(34.0), 219(70.5), 249(85.8), 277(9.8), 333(33.6)
8	Fuc	2219	73(100), 117(81.9), 135(28.7), 147(37.4), 219(65.6), 249(82.1), 277(7.57), 333(25.7)
9	GalUA	2295	73(53.7), 135(100), 103(3.5), 147(9.8), 217(21.9), 305(6.7), 361(1.2)
10	GlcUA	2334	73(97.8), 135(100), 103(11.7), 147(27.6), 217(67.8), 305(33.5), 361(4.5)
11	Glc	2359	73(100), 103(41.7), 147(57.8), 205(51.0), 217(77.8), 307(47.6), 319(59.4), 331(7.7)
12	Man	2364	73(100), 103(41.5), 135(31.2), 147(51.9), 205(38.1), 217(46.9), 249(84.3), 307(48.3), 319(58.7), 331(7.5)
13	Gal	2388	73(100), 103(43.5), 147(58.7), 205(49.1), 217(53.6), 249(85.2), 307(30.9), 319(58.1), 331(7.9)

**Table 2:** Calibration curves and detection limit of thirteen monosaccharides based on as their TMSDs.

No.	TMSDs	Regression equations	*R* ^2^	Linear ranges (μg/mL)	LOD (μg/mL)	LOQ (μg/mL)
1	GlcN	*y* = 0.0229*x* + 0.0062	0.9923	16.3–650.8	1.3	6.5
2	GalN	*y* = 0.0232*x* + 0.0572	0.9951	16.5–659.2	1.3	6.6
3	ManN	*y* = 0.0285*x* + 0.0888	0.9975	16.3–652.7	1.3	6.5
4	Fru	*y* = 0.0103*x* + 0.0412	0.9921	33.2–664.2	2.7	13.3
5	Xyl	*y* = 0.0249*x* + 0.1878	0.9920	8.4–336.5	0.7	3.4
6	Ara	*y* = 0.0133*x* + 0.0874	0.9928	7.7–306.6	0.6	3.1
7	Rha	*y* = 0.0159*x* + 0.1246	0.9924	16.8–672.1	1.3	6.7
8	Fuc	*y* = 0.0163*x* + 0.1314	0.9921	16.9–676.2	1.4	6.8
9	GalUA	*y* = 0.0243*x* + 0.0131	0.9961	20.8–415.2	1.7	8.3
10	GlcUA	*y* = 0.026*x* + 0.1254	0.9933	20.5–410.3	1.6	8.2
11	Glc	*y* = 0.018*x* + 0.1778	0.9921	10.1–402.9	0.8	4.0
12	Man	*y* = 0.0188*x* + 0.1396	0.9933	10.2–407.5	0.8	4.1
13	Gal	*y* = 0.0176*x* + 0.1269	0.9920	10.1–403.3	0.8	4.0
